# Effects of a Large Dose of Quinine Administered by Mistake

**Published:** 1844-03-09

**Authors:** John M. Deere


### EFFECTS OF A LARGE DOSE OF QUININE ADMINISTERED BY MISTAKE.

By John M. Deere, M. D.

In November, 1841, a young lady, aged eighteen, of a delicate constitution and nervous temperament, was under the treatment of a medical friend of mine for severe hysterical symptoms. Almost every evening she had repeated fits—epileptic in appearance, although decidedly hysterical in character. The remedy administered was quinine, in combination with sulphuric acid; she began with six grains per diem, which was soon increased to ten, and afterwards to twenty. When she had been taking the medicine for about a fortnight, she received a six ounce bottle, containing six drachms of quinine in solution, with an ounce and a half of the dilute sulphuric acid; of which she was directed to take one teaspoonful at a dose, in a wineglassful of water. Not regarding the directions, however, she poured out a wineglassful—about one-third of the bottle—and swallowed it.

Her first impression was one of extreme acidity of the mouth, and a most disagreeable sensation about the teeth. This was followed by nausea and extreme giddiness, and tendency to stupor. Her friends, believing that she had taken a narcotic poison, insisted on making her walk up and down, after the manner generally recommended for narcotic poisoning. After this had been kept up some time, the stupor abated, and she passed into a state of semi-consciousness, with the feeling as if she was obliged to keep moving in spite of herself. She was intensely thirsty, and drank a great deal of water. But all the bad symptoms gradually subsided without medical aid, and the epileptic fits, which were the occasion of the treatment, did not appear for a very long time afterwards. Her catamenia were brought on immediately.—*Prov. Med. Jour.*, Dec. 23, 1843.

